# Multi-Omics Analysis After Vaginal Administration of *Bacteroides fragilis* in Chickens

**DOI:** 10.3389/fmicb.2022.846011

**Published:** 2022-02-16

**Authors:** Lu Chen, Maosen Yang, Wei Zhu, Yuan Su, Diyan Li, Tao Wang

**Affiliations:** ^1^Farm Animal Genetic Resources Exploration and Innovation Key Laboratory of Sichuan Province, Sichuan Agricultural University, Chengdu, China; ^2^School of Pharmacy, Chengdu University, Chengdu, China

**Keywords:** chicken, vagina, cloaca, *Bacteroides fragilis*, microbiota, transcriptome, metabolome

## Abstract

The reproductive tract of chickens is an important organ for egg formation. The vagina is in close contact with the external environment, which may lead to the invasion of a variety of pathogenic bacteria, affect the internal and external quality of eggs, and even increase mortality and cause economic loss. In recent years, probiotics as a substitute for antibiotics have brought economic benefits in livestock and poultry production. In the present study, we investigated the effects of vaginal administration of *Bacteroides fragilis* on the cloacal microbiota, vaginal transcriptome and metabolomics of chickens and evaluated the beneficial potential of *B. fragilis.* The results showed that *B. fragilis* treatment could affect the microbial composition of the cloaca. Transcriptome analysis found that the immune-related genes *CCN3*, *HAS2*, and *RICTOR* were upregulated, that the inflammatory genes *EDNRB*, *TOX*, and *NKX2-3* were downregulated, and that DEGs were also enriched in the regulation of the inflammatory response, cellular metabolism, and synaptic response pathways. In addition, the differential metabolites were mainly related to steroid hormone biosynthesis, unsaturated fatty acid biosynthesis, and arachidonic acid metabolism, and we identified associations between specific differential metabolites and genes. Overall, this study provides a theoretical basis for the application of *B. fragilis* as a potential probiotic in livestock and poultry production.

## Introduction

The hen’s reproductive tract is not as abundant in mammals; it is mainly used for egg formation (Sah and Mishra, 2018). Some pathogenic bacteria colonization of the oviduct can lead to egg pollution, such as *Salmonella strains* and *Gallibacterium anatis* (McWhorter and Chousalkar, 2016; Zhang et al., 2017). Therefore, the prevention of reproductive tract infection by pathogens is important for the safe production of eggs and the health of chickens. In recent years, with the rise of drug-resistant strains and adverse reactions after the use of antibiotics, probiotics have gradually entered poultry production, which can improve intestinal health, prevent disease, and promote nutrient absorption (Shini et al., 2013; Jeni et al., 2021).

*Bacteroides fragilis* is a gram-negative obligate anaerobe that often colonizes the oral cavity, intestinal tract, and female reproductive tract. A nontoxic *B. fragilis* (NTBF) strain uses dietary and host-derived polysaccharides as a source of carbon and energy (Spence et al., 2006). Moreover, *B. fragilis* has been shown to inhibit inflammation in the intestinal tract and cancer associated with enteritis (Lee et al., 2018; Zheng et al., 2020; Shao et al., 2021); it also participates in the immunomodulatory regulation of brain immunity through the gut-brain axis (Erturk-Hasdemir et al., 2021) and regulates inflammation in other areas (Johnson et al., 2018). The strain also suppressed pathogen infection, such as *Vibrio parahaemolyticus* and *Salmonella Heidelberg* (Li et al., 2017; Vernay et al., 2020). Although *B. fragilis* performed a variety of beneficial functions in other hosts, its effects in chickens remains unclear.

With the development of high-throughput sequencing technology, many studies are being conducted on multi-omics analysis. Previous studies have found that diet supplementation with the combination of *Bacillus* species for aging laying hens can reduce the number of *Escherichia coli* and increase *Lactobacillus* in the cecum (Yang et al., 2020). In addition, Wang et al. (2019b) found that oral administration of *Lactobacillus frumenti* can regulate lipid and amino acid metabolism and promote liver energy production in early weaned piglets. Recently, Chen et al. (2021) conducted a gavage experiment using germ-free mice that activated TLR4 and mTOR signaling pathways and increased the expression of fat-accumulating genes, leading to host inflammation. Although probiotics are generally treated using subcutaneous injection and intragastric administration, vaginal administration has been used to better study the effect of probiotics on the reproductive tract in recent years (Deng et al., 2014; Genís et al., 2017). Studies have found that using probiotics in the vagina can reduce the incidence of vaginal infections in women (Deng et al., 2014). In poultry, lactic acid bacteria by vaginal administration can safely colonize the vagina, eliminate some pathogens, and modulate the immune response (Reid and Bocking, 2003). Therefore, we attempted to explore the effects of intravaginal administration of *B. fragilis* on the microbiome, transcriptome, and metabolome of the chicken reproductive tract in this study.

## Materials and Methods

### Bacterial Strains and Culture Conditions

*Bacteroides fragilis* ATCC25285 was obtained from the American Type Culture Collection (Manassas, VI, United States). The strain was anaerobically cultured on tryptic soy agar (TSA) supplemented with 5% sheep blood at 37°C under anaerobic workbench conditions containing 80% N_2_, 10% CO_2_, and 10% H_2_. After purification and 16S rRNA gene sequencing identification, collected strains were prepared with cell pellets resuspended in phosphate-buffered saline (PBS) supplemented with 20% glycerol after centrifugation and preserved at −80°C. The bacterial solution was colony-forming unit (cfu) enumerated on TSA with 5% sheep blood before injection.

### Animals and Experimental Design

This experiment was conducted in the poultry breeding base of Sichuan Agricultural University in Ya’an, China. Eleven 320-day-old green shell laying hens with the same weight and diet without antibiotics were randomly divided into a control group (*n* = 5) and a *B. fragilis* group (*n* = 6). The *B. fragilis* or vehicle (sterile PBS with 20% glycerin) were infused into the vaginal tract gently with 3-ml sterile pasteur pipettes (NEST, Wuxi, China). The control group was given 500 μl sterile PBS with 20% glycerin. The *B. fragilis* group was given 500 μl (1×10^10^ CFU/ml) of *B. fragilis* suspension by intravaginal administration. Injections were given every other day for a total of four times (Hsiao et al., 2013). Cloaca swabs were collected with cotton swabs after observation for 2 months (Mazmanian et al., 2005). Then, all chickens were sacrificed by cervical dislocation. Vaginal tissue samples were sampled from each experimental chicken for further RNA-seq and metabolome analysis. Storing all collected samples in a −80°C refrigerator.

### DNA Extraction and Microbial 16S rRNA Sequencing Analysis

Cloacal microbial DNA was extracted using the Ezup Oral Swabs Genomic DNA Extraction Kit (Sangon Biotech, Shanghai, China) following the manufacturer’s guidelines. The extracted DNA was quantified using a NanoDrop2000 spectrophotometer (Thermo Fisher Scientific, DE, United States), and the quality was assessed by 1% agarose gel electrophoresis. The V3-V4 hypervariable regions of the bacterial 16S rRNA gene were amplified used specific primer 341F (5′-CCTAYGGGRBGCASCAG-3′) and 806R (5′-GGACTACNNGGGTATCTAAT-3′). All PCRs were carried out with 15 μl of Phusion® High-Fidelity PCR Master Mix (New England Biolabs), 0.2 μM forward and reverse primers, and approximately 10 ng template DNA. Thermal cycling consisted of the initial denaturation at 98°C for 1 min, followed by 30 cycles of denaturation at 98°C for 10 s, annealing at 50°C for 30 s, and elongation at 72°C for 30 s, and then, finally 72°C for 5 min. PCR products were purified with a Qiagen Gel Extraction Kit (Qiagen, Germany). Sequencing libraries were generated using the TruSeq® DNA PCR-Free Sample Preparation Kit (Illumina, United States) following the manufacturer’s recommendations, and index codes were added. The library quality was assessed on the Qubit@2.0 Fluorometer (Thermo Scientific) and Agilent Bioanalyzer 2100 system. Finally, the library was sequenced on an Illumina NovaSeq platform, and 250 bp paired-end reads were generated.

The raw 16S rRNA gene sequencing data were processed using QIIME2 (Bolyen et al., 2019). Then, these data were filtered to eliminate adapters and low-quality reads for clean reads. The high-quality representative feature sequences were used as the reference for taxonomic annotation with the Silva database (Quast et al., 2013).^1^ Alpha diversity is represented by faith_ pd and evenness indices, and different groups were compared using the Kruskal–Wallis test. Principal coordinates analysis (PCoA) is represented by unweighted UniFrac distance, and PERMANOVA was used for intergroup significance tests.

### RNA Extraction and Transcriptome Analysis

Total RNA was extracted from vaginal tissue using RNAiso Plus Total RNA extraction reagent (Takara) following the manufacturer’s instructions, and RNA was detected with a NanoDrop2000 spectrophotometer (Thermo Fisher Scientific, DE, United States). The quantity of total RNA was determined using a Qubit 2.0 fluorimeter (Life Technologies, CA, United States), and the integrity of total RNA was analyzed using a Bioanalyzer 2100 system (Agilent Technologies, CA, United States). After the RNA samples were qualified, the eukaryotic mRNA was enriched by magnetic beads with oligo(dT). Then, cDNA was synthesized using mRNA as template. Second-strand cDNA was synthesized by buffer, dNTPs, DNA polymerase I and RNase H and purified using AMPure XP Beads. The purified double-stranded cDNA was end repaired; poly(A) was added, and the cDNA was ligated to a sequencing connector. After fragment selection and PCR amplification, a sequencing library was obtained and sequenced using DNBSEQ-T7 by Novogene (Beijing, China).

We removed low-quality reads and calculated the Q20, Q30, and GC contents of the clean reads to obtain high-quality clean reads. Clean data were mapped to the chicken reference genome (Gallus-6.0) using the STAR alignment tool (V2.7.6a). Then, Kallisto (V0.44.0) software was used to quantify gene expression as transcripts per million (TPM). Benjamini and Hochberg’s procedure was used to adjust the value of *p* (Audic and Claverie, 1997; Benjamini and Yekutieli, 2001). The differentially expressed genes (DEGs) with an adjusted value of *p*(*P*adj) < 0.05 and | log2 (fold change) | > 1 identified by DESeq2 (v1.30.1) (Wang et al., 2010) were considered as differentially expressed. Functional enrichment including GO terms and KEGG pathways of differentially expressed genes was performed by Metascape (Zhou et al., 2019b), and *p* < 0.05 was considered as significant. Then, 10 randomly selected DEGs were used to verify the transcriptome results by quantitative real-time PCR (qRT–PCR). Total RNA was reverse transcribed into cDNA using the RT Easy™ II (with gDNase) kit (FOREGENE, Chengdu, China). Next, RT–qPCR amplification was performed using SYBR Green Master Mix, and the chicken β-actin gene was used as a housekeeping gene. The relative expression levels of validated genes were determined using the 2^-△△Ct^ method. Primer sequences are listed in Supplementary Table 1.

### Metabolomic Analysis

Metabolic analysis was performed on vaginal tissue used in this study. Metabolome extraction and pretreatment were based on protocols followed by Novogene (Beijing, China) and mainly used liquid mass spectrometry (LC–MS) technology (Want et al., 2010; Dunn et al., 2011). After qualitative and quantitative analysis of metabolites, data quality control was carried out to ensure the accuracy and reliability of the data results. Then, multivariate statistical analysis, including principal component analysis (PCA) and partial least squares discriminant analysis (PLS-DA), was carried out for metabolites. The two parameters of variable importance in the projection value (VIP) > 1 and *p* < 0.05 were used as the criteria to screen differential metabolites. Finally, the biological significance of metabolites was explained by the functional analysis of metabolic pathways based on the Kyoto Encyclopedia of Genes and Genomes (KEGG) database.^2^

### Transcriptome and Metabolome Association Analysis

To analyze the correlation between the relative abundance of specific DEGs and significantly different metabolites, the correlation coefficients rho and value *p* were calculated by Pearson’s statistical methods.

## Results

### Effects of the Vaginal Administration of *Bacteroides fragilis* on Cloacal Microbiota

**Figure 1:**
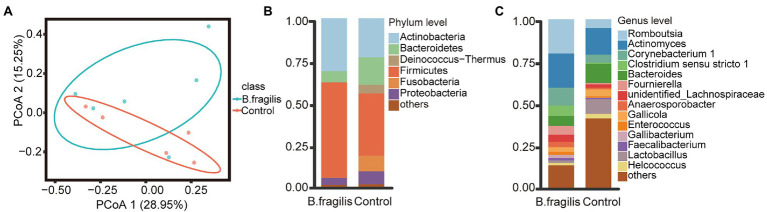
Composition and distribution of the cloacal microbiota after the intravaginal administration of *Bacteroides fragilis* between the *Bacteroides fragilis* and control groups in chickens. **(A)** Principal coordinate analysis (PCoA) of bacterial community structures using the unweighted UniFrac method. **(B)** The relative abundances of the top seven phyla from cloaca samples. **(C)** The relative abundances of the top 15 genera in the microbiota.

We performed 16S rRNA sequencing on the cloacal microbiota. After quality control, up to 982,622 high-quality reads were generated from 11 samples with an average of 89,329 reads per sample (Supplementary Table 2). We then used evenness and faith_ pd indices to visualize the microbiota diversity in different groups. There was no significant alpha diversity in the *B. fragilis* group and control group (Kruskal–Wallis, *p* > 0.05), but principal coordinate analysis (PCoA) based on unweighted UniFrac distance showed a trend of separation, although the difference was not significant (Supplementary Figure 1; Figure 1A).

Next, we studied changes in the microbial composition of the two groups. At the phylum level (relative abundance >1%), Firmicutes, Actinobacteria, Proteobacteria, and Bacteroidetes were dominant phyla in the groups. However, Firmicutes and Actinobacteria represented a higher proportion of the phyla in the *B. fragilis* group, with relative abundances of 56.45 and 31.37%, respectively. Bacteroidetes (16.34%), Fusobacteria (9.25%), and Proteobacteria (7.72%) were more abundant in the control group (Figure 1B). At the genus level (relative abundance > 0.05%), taxa unclassified below the family level (43.14%) were the most abundant in the control group, but the most dominant genus in the *B. fragilis* group was *Romboutsia*, with a relative abundance of 20.38%, followed by *Actinomyces* (20.26%), *Corynebacterium 1* (10.62%), *Clostridium sensu stricto 1* (6.08%), *Fournierella* (5.14%), *unidentified_Lachnospiraceae* (4.31%), and *Anaerosporobacter* (3.12%), the abundance of these genera were higher than that of the control group (Figure 1C).

### Transcriptome Analysis of Vagina Tissues After *Bacteroides fragilis* Treatment

To identify differentially expressed mRNAs of vaginal tissue in the control group and *B. fragilis* group, we carried out transcriptome sequencing. The results of transcriptome sequencing and quality parameters are shown in Supplementary Table 3. A total of 153.24 G of raw data was produced in this sequencing, and 149.69 G of clean data was obtained after filtration. The GC content of clean samples ranged from 47.89% to 50.32%, and the average percentage of Q20 bases in each sample was 95.53%. The high-quality reads were mapped to the chicken reference genome, and the average mapping was 95.78%.

**Figure 2:**
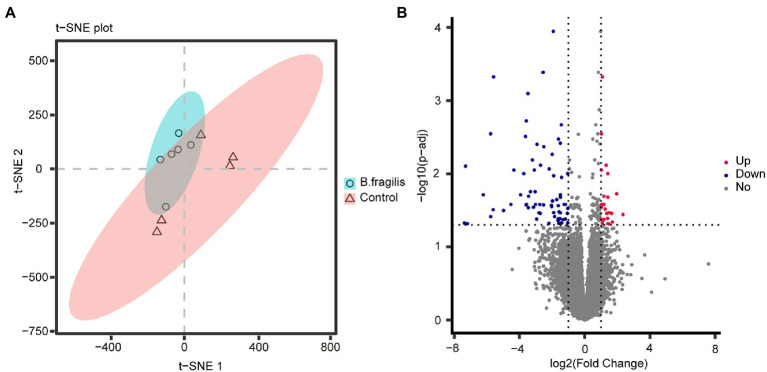
Transcriptomic analysis of vaginal tissue between the *Bacteroides fragilis* group and the control group. **(A)** t-distributed stochastic neighbor embedding (t-SNE) based on gene expression as transcripts per million (TPM). **(B)** Volcano map of differentially expressed genes between the two groups.

**Figure 3:**
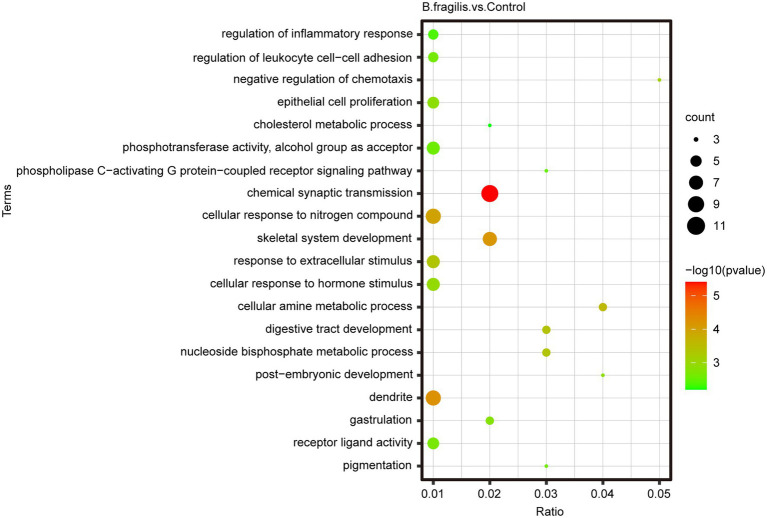
Top 20 most enriched gene ontology (GO) terms of differentially expressed genes (DEGs). The X-axis shows the proportion of enriched genes to total genes in this term; the Y-axis shows the DEG-enriched GO term. The size and color of dots represent the number of genes and the value of *p*.

Correlation analysis between samples was performed based on gene expression levels. Spearman correlation analysis showed that the 11 biological repeat samples were clustered, indicating that the sample selection was relatively reasonable (Supplementary Figure 2). t-SNE result revealed that there were significant differences between the *B. fragilis* and control groups (Figure 2A). A total of 16,776 genes were detected in both groups. Then, we screened 94 differentially expressed genes (DEGs) using *P*adj < 0.05 and | log2 (fold change) | > 1 as the standards, including 26 upregulated genes and 68 downregulated genes (Figure 2B). Then, we performed GO and KEGG enrichment analyses on these DEGs to study their biological functions. Figure 3 shows the top 20 most enriched GO terms related to DEGs. The pathways related to immunity or inflammatory response terms mainly included the regulation of leukocyte cell–cell adhesion, the negative regulation of chemotaxis, and the regulation of inflammatory response and epithelial cell proliferation. The major DEGs involved in these GO terms included *CCN3*, *HAS2*, *RICTOR*, *TOX*, *EDNRB*, and *NKX2-3*. The remaining GO terms mainly included the metabolic process and cell and synaptic reactions, such as cellular response to hormone stimulus, cellular response to nitrogen compound, nucleoside bisphosphate metabolic process and cholesterol metabolic process, and chemical synaptic transmission. KEGG pathways enriched by DEGs mainly included the MAPK signaling pathway, calcium signaling pathway, and rheumatoid arthritis (Supplementary Table 4).

To validate the results of RNA-seq analysis, a total of 10 DEGs, including *CCN3*, *CTLA4*, *DIO2*, *ITPKA*, *PDK4*, *RICTOR*, *MYO5A*, *NPY*, *SEMA3c*, and *SYT12*, were subjected to quantitative reverse transcription-PCR (RT–qPCR; Supplementary Table 5). We found that the expression trend of RT–qPCR was consistent with the transcriptome through calculation, indicating the reliability of RNA-seq data.

### Metabolic Profiling of Vagina Tissues Following the Intravaginal Administration of *Bacteroides fragilis*

**Figure 4:**
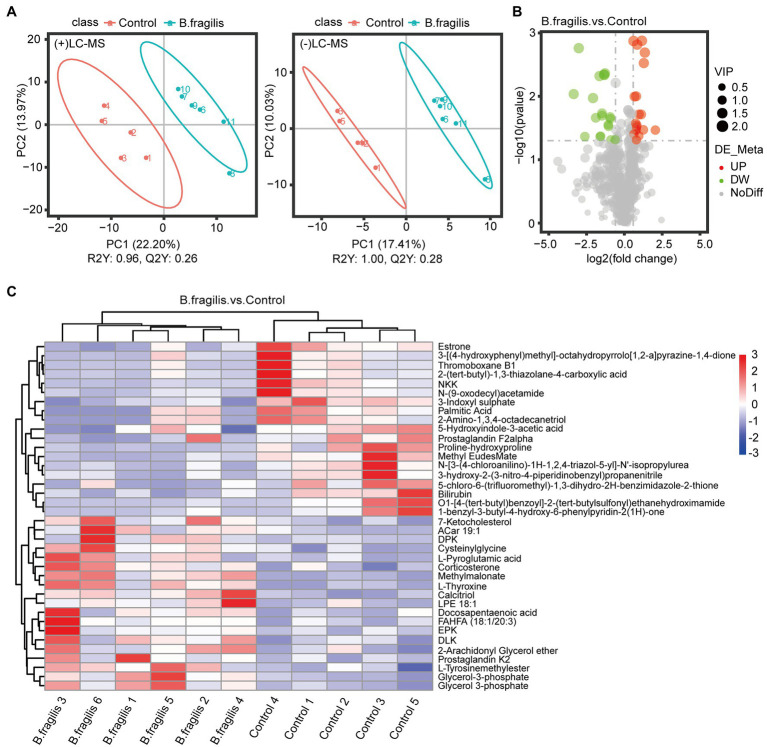
Metabolomics analysis of vaginal differential metabolites between the two groups. **(A)** Score scatter plot of vaginal metabolites in the control and *Bacteroides fragilis* groups with *Bacteroides fragilis* treatment by PLS-DA in positive mode and negative mode. **(B)** Volcano plot of the differential metabolites. **(C)** The differential metabolite clustering heatmap. Each column represents a sample, and each row represents a metabolite.

First, PLS-DA pattern analysis was performed on the control and *B. fragilis* groups to identify the overall metabolic differences between the two groups. According to the results in Figure 4A, we observed a significant trend toward separation of two groups in positive ion mode and negative ion mode. In addition, PCA showed excellent separation of metabolites between the two groups (Supplementary Figure 3).

**Figure 5:**
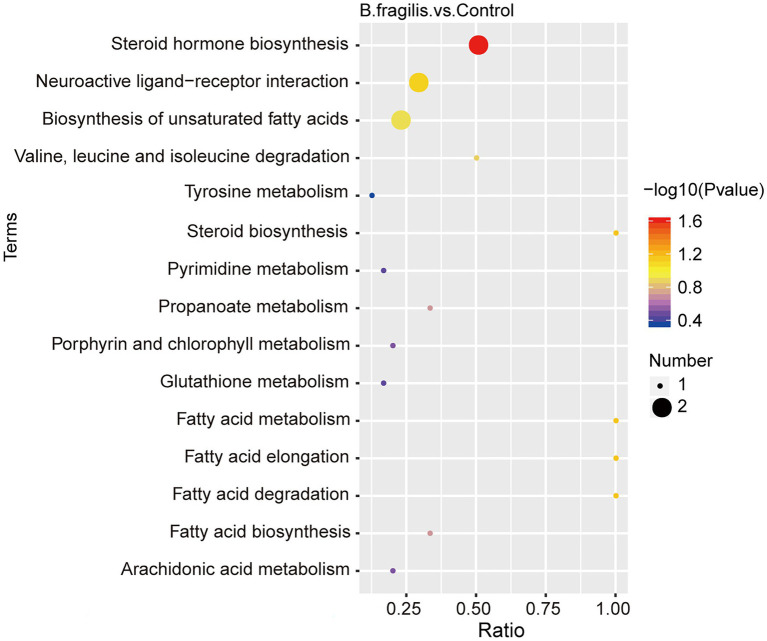
KEGG pathway analysis of the altered metabolites in the vagina. The color of the points represents the value of *p* of the hypergeometric test, and the size of the points represents the number of differential metabolites in the corresponding pathway.

The metabolomic analysis identified 38 metabolites with significant differences between the control and *B. fragilis* groups based on the value of VIP > 1.0 (*p* < 0.05). Among these metabolites are nine lipids and lipid-like molecules; the levels of these, except for estrone, palmitic acid, and prostaglandin F2alpha decreased; calcitriol, glycerol-3-phosphate, 7-ketocholesterol, corticosterone, 2-arachidonyl glycerol ether, and docosapentaenoic acid increased in the *B. fragilis* group compared with the control group. There were also eight organic acids and derivatives (Supplementary Table 6). At the same time, changes in these metabolites are shown in a volcano map and a heatmap generated by hierarchical clustering analysis (Figures 4B,C). Next, we used KEGG pathway enrichment to identify the most important biochemical metabolic pathways and signal transduction pathways involved in differential metabolites. Pathway enrichment analysis showed that the intravaginal administration of *B. fragilis* resulted in changes in lipid metabolism, including steroid hormone biosynthesis; the biosynthesis of unsaturated fatty acids; steroid biosynthesis; arachidonic acid metabolism; and the elongation, degradation, metabolism, and biosynthesis of fatty acids. The bacterial strain also regulated valine, leucine, and isoleucine degradation, tyrosine metabolism, and propanoate metabolism pathways (Figure 5).

### Combined Analysis of Specific Differential Genes and Metabolites

**Figure 6:**
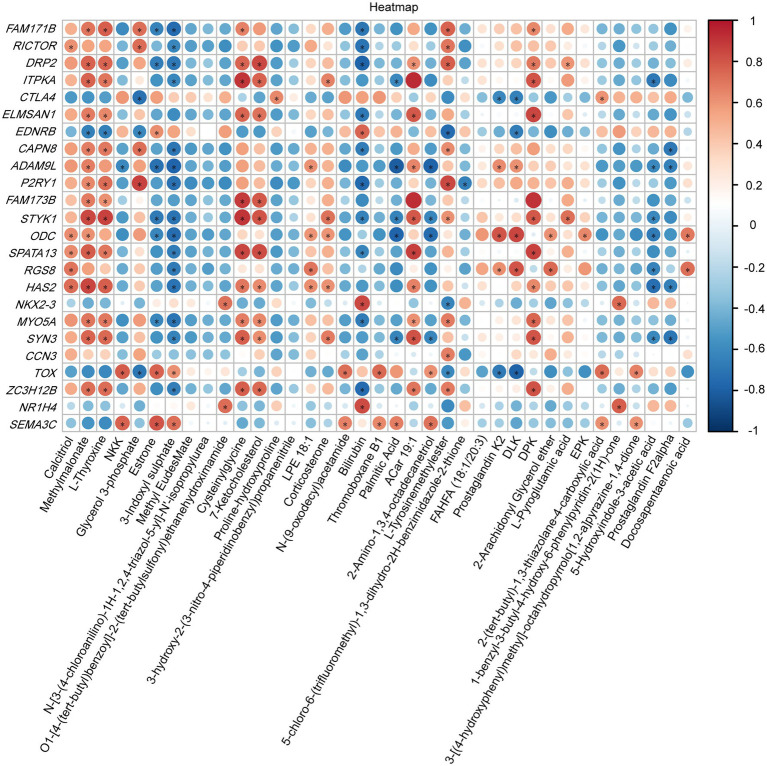
Heatmap of specifically differentially expressed genes significantly associated with differential metabolites, as determined by Pearson’s correlation analysis (^*^p < 0.05).

To investigate the relationship between the vaginal transcriptome and metabolome after treatment with *B. fragilis*, we performed Pearson correlation analysis based on 24 differential genes and 37 differential metabolites. Calcitriol, methylmalonate, l-thyroxine, cysteinylglycine, and 7-ketocholesterol were positively correlated with most DEGs, such as *DRP2*, *ITPKA*, *STYK1*, and *HAS2* but were negatively correlated with *EDNRB*. Estrone, 3-indoxyl sulfate and bilirubin showed the opposite results, which were significantly negatively correlated with a majority of differential genes (Figure 6).

## Discussion

The chicken oviduct is an organ that receives ovulation from the ovary and completes egg formation; the vagina is located in the lower part and contacts the environment (Abdel-Mageed et al., 2017). Hence, chicken vagina is easily infected by pathogenic bacteria, resulting in salpingitis, peritonitis and other reproductive tract diseases, which leads to lower egg production and even increased mortality (Bojesen et al., 2004; Allahghadry et al., 2021). Recent studies have shown that reproductive tract microbiota is the key factor affecting reproductive tract health and production performance of livestock and poultry. *Bacteroidetes* are the main bacterial communities in reproductive tract, among *B. fragilis ATCC 25285* (NCTC 9343) was one of the earliest to show beneficial effects on the host and played a vital role by releasing the main functional molecule polysaccharide A (PSA) (Tan et al., 2019). Our previous studies have shown that *B. fragilis* is significantly associated with a higher egg production. It may be that the immune system regulates reproductive activity in birds (Su et al., 2021). Similarly, the results of multiple omics in this study suggested that *B. fragilis* does affect immune function and metabolic homeostasis in the reproductive tract of chickens.

In this study, cloacal swabs were used for microbial analysis. The study found that the oviduct microbial composition is similar in chicken, and commensal bacteria from hens may be vertical transferred to the embryo (Lee et al., 2019). Moreover, intestinal tract and reproductive tract composition of microbiomes have a large overlap. This is mainly due to the physical results of chicken, the cloaca of chicken connects the intestinal tract and the reproductive tract (Shterzer et al., 2020). These findings suggested that the vagina and cloacal microbes may also communicate with each other.

Previous studies have shown that the oral administration of *B. fragilis* improves the offspring in a maternal immune activation (MIA) model in mouse intestinal barrier integrity and microbial composition (Hsiao et al., 2013). In this study, no significant differences were observed following the *B. fragilis* treatment of the vagina in alpha diversity or in PCoA. However, vaginal administration altered the composition of the cloacal microbes, and the relative abundances of *Clostridium sensu stricto 1* and *Romboutsia* in the *B. fragilis* group were relatively high. Specific *Clostridium* has the potential to synthesize antibacterial compounds (Pahalagedara et al., 2020). Zhou et al. (2019a) found that *Clostridium butyricum WZ001* inhibited inflammation induced by *E. coli* and maintained vaginal microecological balance in mice. In addition, *Romboutsia* had the highest proportion in the *B. fragilis* group. A recent study showed that *Romboutsia* can be used as a predictor of egg production in chickens and that it reduces proinflammatory cytokines in the serum (Liang et al., 2016; Wen et al., 2021). These results suggest that *B. fragilis* is able to collectively improve reproductive tract health by altering microbial composition.

Vaginal transcriptome analysis showed that DEGs were enriched in inflammatory response regulation, regulation of leukocyte cell–cell adhesion, metabolic process, cellular response, and synaptic transmission. Our study indicated that the immune-related genes *CCN3*, *HAS2*, and *RICTOR* were upregulated in the *B. fragilis group*. *CCN3* participates in the functional regulation of regulatory T cells (Tregs) and hematopoietic stem cells; it can regulate angiogenesis and promote endothelial cell adhesion and survival (Lin et al., 2003; Peng et al., 2021). Studies have shown that *CCN3* loss leads to a significant increase in lipid uptake and foam cell formation in macrophages, while overexpression can inhibit atherosclerosis (Shi et al., 2017). Hyaluronan Synthase 2 (*HAS2*) has a protective effect on airway inflammation and emphysema induced by elastase in mice (Osawa et al., 2020). *RICTOR* has been found to play an important role in cell autophagy and metabolism (Zhao et al., 2020). Xu et al. (2018) demonstrated that enhanced *RICTOR* expression may reduce liver injury inflammation. This is consistent with our study showing that the high expression of this gene may influence vaginal cell metabolic and pathway responses.

In addition, genes associated with inflammation, such as *EDNRB*, *TOX*, and *NKX2-3*, were downregulated in the *B. fragilis group*. *EDNRB* is related to intestinal mucosal inflammation, and probiotic treatment can downregulate the mRNA expression of this gene and exert anti-inflammatory effects by reducing macrophages and dendritic cells (Plaza-Díaz et al., 2017). *TOX* is a transcription factor in cancer progression, and it has been shown that the downregulation of *TOX* in CD8+ T cells can enhance the antitumor effect of cells (Wang et al., 2019a). *NKX2-3* is a transcription factor associated with inflammatory bowel disease (IBD), and its expression is increased in Crohn’s disease (CD; Yu et al., 2012). Overall, these results suggest that *B. fragilis* treatment may exert its protective effect by upregulating immune genes and downregulating inflammation-related genes.

In the analysis of the metabolome of the intravaginal administration of *B. fragilis*, we found that there were significant differences in the differential metabolites between the *B. fragilis* group and the control group, including nine lipid-related molecules. Calcitriol is the active metabolite of vitamin D3; it has shown an anti-inflammatory effect on human corneal epithelial cells infected with *Pseudomonas aeruginosa* and suppresses the production of TNF-α and IL-1α (Kernacki and Berk, 1994; Xue et al., 2002). Meanwhile, calcitriol significantly inhibited the expression of NLRP3 inflammasome-related genes and IL-1β production in hyperosmotic stress (HS)-exposed cells (Dai et al., 2019). In addition, the combination of progesterone and calcitriol significantly inhibited the growth of endometrial and ovarian cancer cells (Paucarmayta et al., 2020). The results of our study showed that calcitriol levels in the *B. fragilis group* were significantly higher than those in the control group, and vaginal administration also significantly altered steroid and steroid hormone biosynthesis according to KEGG. Steroid hormones include sex hormones and adrenal corticosteroids, which regulate innate and adaptive immunity and play an important role in reproduction (Czyzyk et al., 2017; Moulton, 2018; Cutolo and Straub, 2020).

Most of the differential metabolites were enriched in the metabolic pathways of fatty acids, including the biosynthesis of unsaturated fatty acids, arachidonic acid metabolism and various pathways of fatty acids. Arachidonic acid and docosapentaenoic acid are unsaturated fatty acids. Arachidonic acid is an essential dietary fatty acid that exists in the form of esterification in structural phospholipids in cell membranes throughout the body (Calder, 2007). In humans and other mammals, different enzymes cause membrane arachidonic (ARA) oxidation, resulting in the production of many proinflammatory and anti-inflammatory breakdown mediators (Hyde and Missailidis, 2009; Hanna and Hafez, 2018). In addition, docosapentaenoic acid (DPA) is more likely to be incorporated into inflammatory cells, resulting in a decrease in the synthetic substrates of pro-inflammatory eicosanes (PGE2 and LTB4), thereby regulating the production of inflammatory cytokines (Zheng et al., 2019). In this study, the increase in DPA levels induced by *B. fragilis* may be related to the immune regulation of vaginal cells.

On the other hand, *B. fragilis* produces short-chain fatty acids (SCFAs), mainly in the form of propionic acid. Our results showed that *B. fragilis* regulated propionic acid metabolic pathways. This is consistent with previous research, the oral administration of *B. fragilis* was found to significantly increase the concentration of SCFAs in the intestinal contents of *Salmonella*-infected rats, thereby further reducing inflammation and restoring the integrity of the intestinal barrier (Bukina et al., 2018). Furthermore, propionic acid metabolism can also induce the apoptosis of human colon cancer cells and avoid tumor formation (Cruz-Bravo et al., 2014). The production of SCFAs is closely related to the microbial community. Among *unidentified_Lachnospiraceae* and *Faecalibacterium* can produce SCFAs (Ma et al., 2020; Huang et al., 2021), and the abundance of these two genera in the *B. fragilis* group higher than the control group, suggesting that *B. fragilis* may also play a beneficial role by regulating microbial-metabolic processes. *B. fragilis* also regulated vaginal amino acid metabolism, we found that *B. fragilis* treatment modulated the valine, leucine, and isoleucine degradation pathway. Previous studies have shown that *P. copri* increased the concentration of metabolites of valine, leucine, and isoleucine biosynthesis in pig serum, leading to chronic inflammatory responses in the host (Chen et al., 2021). However, the opposite result was obtained in our study. These results suggest that vaginal injection with *B. fragilis* may have a positive effect on inhibiting vaginal inflammation in chickens. The combined transcriptome and metabolome analysis showed that some differential genes were significantly correlated with metabolites, which were consistent with the expression levels in this study. Such as, calcitriol was found to be a metabolite of vitamin D, which in turn is involved in *HAS2* regulation (Narvaez et al., 2020). This suggested that these differential genes and metabolites may interact to maintain the health of chicken reproductive tract after injection of *B. fragilis*. Meanwhile, this provided a reference for further exploring the possible mechanism of action after *B. fragilis* treatment.

## Conclusion

In summary, our study suggested that the vaginal administration of *B. fragilis* could regulate cloacal microbial composition and affect the expression of vaginal immune and inflammatory genes and metabolism-related pathways. These results provide a theoretical basis for the application of *B. fragilis* as a potential probiotic in livestock and poultry production. In addition, the interaction mechanism between specific genes and metabolites needs to be further studied.

## Data Availability Statement

The datasets presented in this study can be found in online repositories. The names of the repository/repositories and accession number(s) can be found at: Bioproject, ID: PRJNA794049 and PRJNA792058.

## Ethics Statement

The animal study was reviewed and approved by the Institutional Animal Care and Use Committee of Sichuan Agricultural University.

## Author Contributions

DL and TW conceived and designed the experiment. LC performed the experiments and wrote the manuscript. MY and WZ performed the bioinformatic analyses. YS participated in experimental design and sampling. All authors reviewed and approved the final manuscript.

## Funding

This work was supported by grant from the Sichuan Science and Technology Program (2019JDTD0009).

## Conflict of Interest

The authors declare that the research was conducted in the absence of any commercial or financial relationships that could be construed as a potential conflict of interest.

## Publisher’s Note

All claims expressed in this article are solely those of the authors and do not necessarily represent those of their affiliated organizations, or those of the publisher, the editors and the reviewers. Any product that may be evaluated in this article, or claim that may be made by its manufacturer, is not guaranteed or endorsed by the publisher.

## Footnotes

^1^ https://www.arb-silva.de/

^2^ https://www.kegg.jp/
