# Supplementary material for: Multi-Omics Analysis After Vaginal Administration of Bacteroides fragilis in Chickens
Source: Front Microbiol. 2022 Feb 16;13:846011. doi: 10.3389/fmicb.2022.846011 (PMC8888936; doi:10.3389/fmicb.2022.846011)
Supplement: Supplementary file 1 [file Data_Sheet_1.ZIP › Supplementary_Material/Supplementary_Material.docx]

Supplementary Material

# Supplementary Figures and Tables

## Supplementary Figures

##
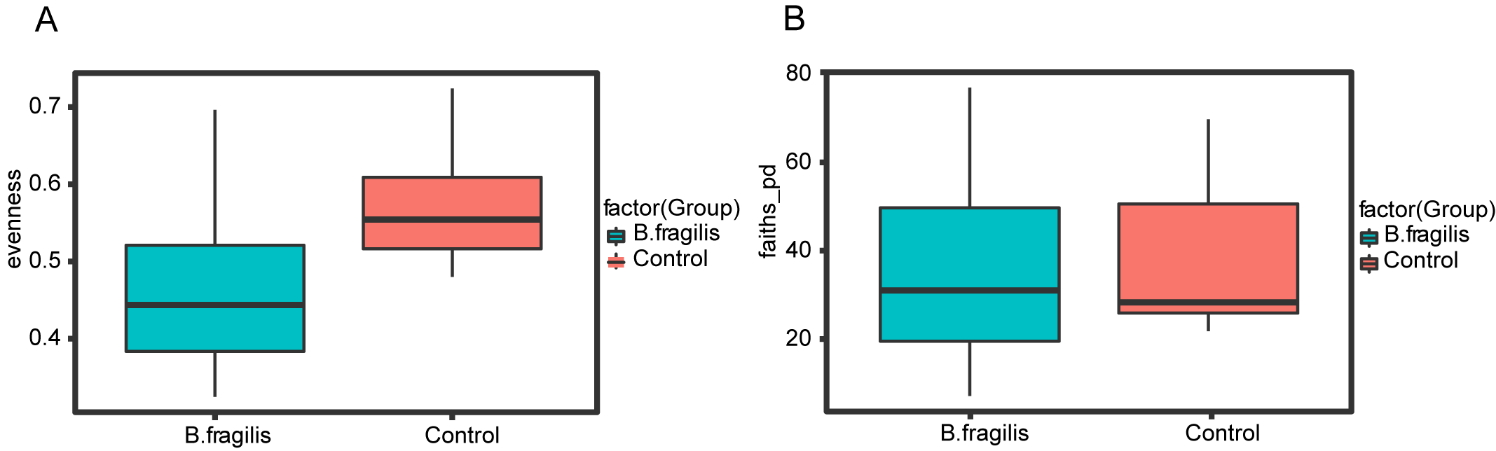
Supplementary Figure 1. Alpha diversity indices of the cloacal microbiota between the two groups. (A) evenness (B) and faith_ pd indices were compared according to the Kruskal–Wallis test to determine significant differences.


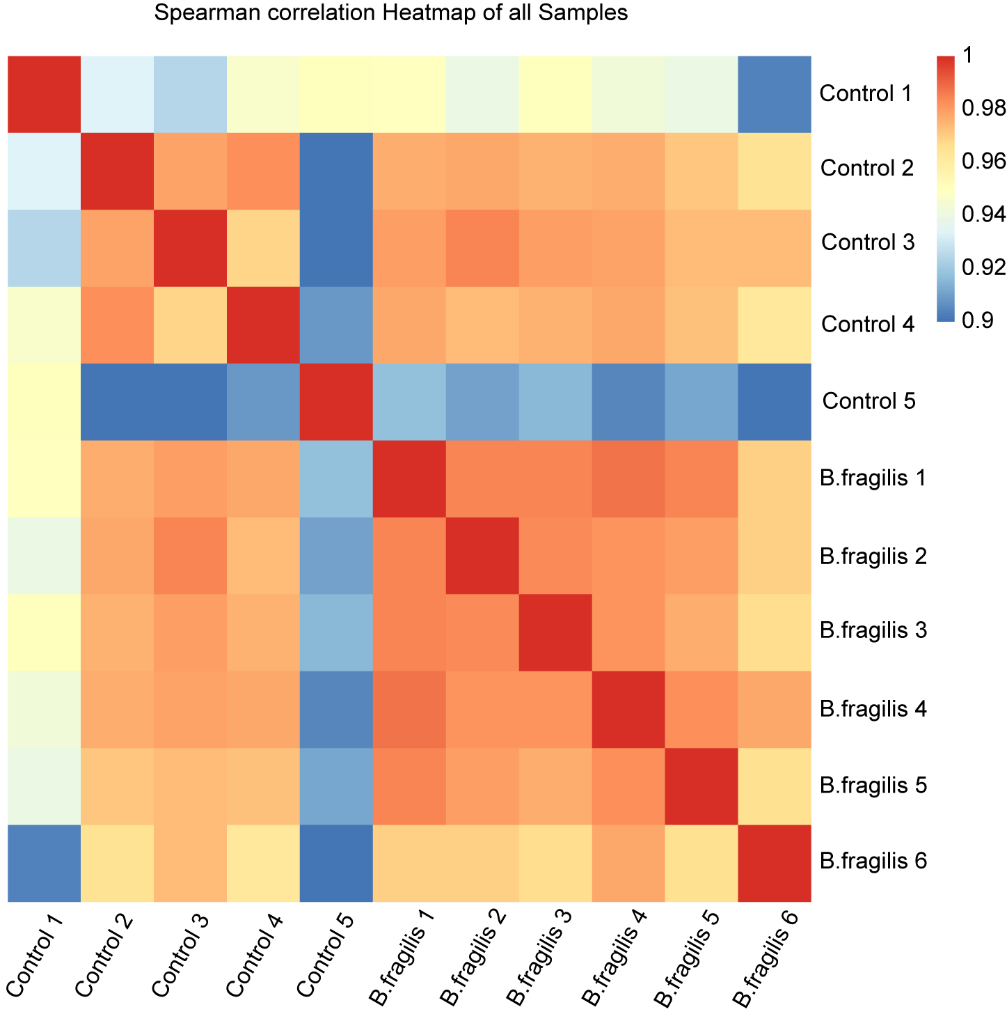


**Supplementary Figure 2**. Spearman’s r heatmap of gene expression in the vagina between the control and *B. fragilis* groups. The color represents the coefficient of association between each pair of samples, and the correlation increases from blue to red.


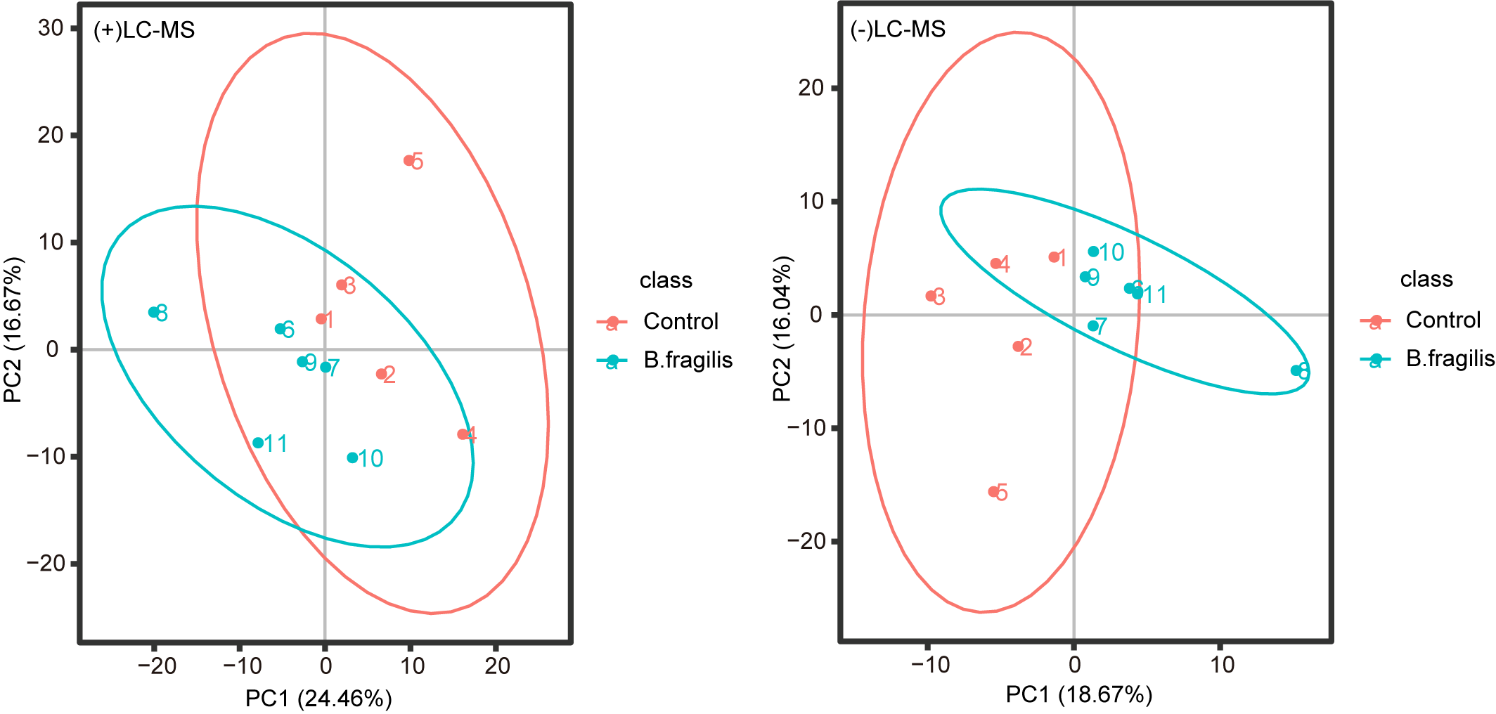


**Supplementary Figure 3**. PCA (principal component analysis) score plot of the control group and the *B. fragilis* group for vaginal tissue in positive mode and negative mode.

## Supplementary Table

**Supplementary Table 1**. Primers for RT-qPCR.

**Supplementary Table 2**. The results of 16S rRNA gene sequencing and quality parameters.

**Supplementary Table 3**. The results of transcriptome sequencing and quality parameters.

**Supplementary Table 4**. Lists of the significantly enriched KEGG pathways associated with vaginal DEGs.

**Supplementary Table 5**. qRT–PCR results of expression data of 10 randomly selected genes by RNA-seq.

**Supplementary Table 6**. Differential vaginal metabolites in chickens in the two groups.
