# Supplementary figures and images for: Multi-Omics Analysis After Vaginal Administration of Bacteroides fragilis in Chickens
Source: Front Microbiol. 2022 Feb 16;13:846011. doi: 10.3389/fmicb.2022.846011 (PMC8888936; doi:10.3389/fmicb.2022.846011)

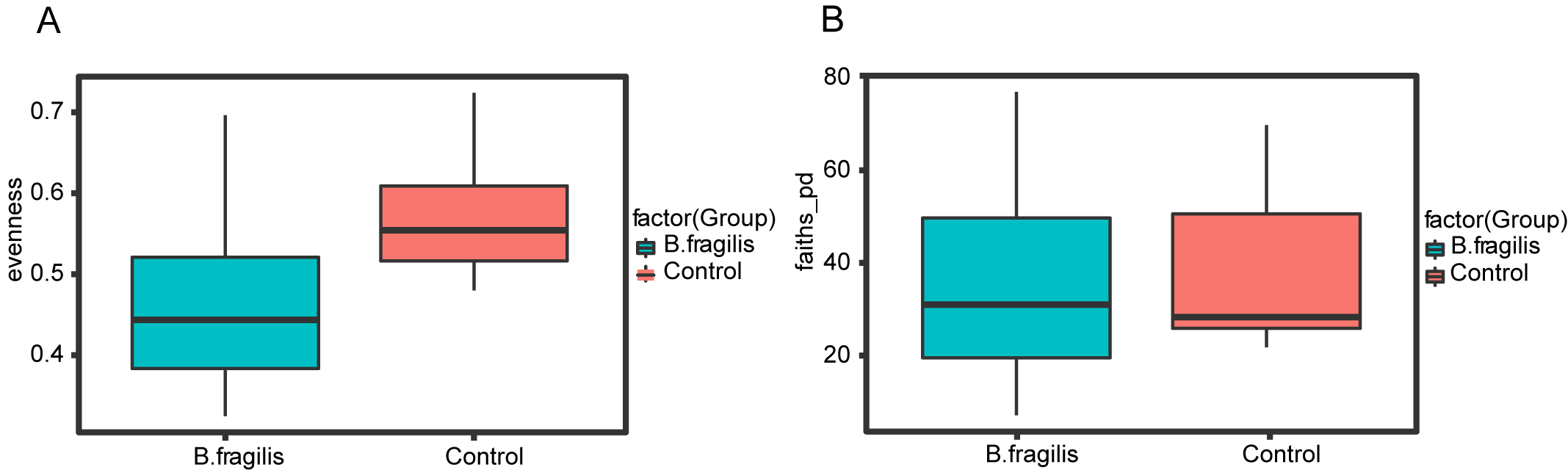

Supplement: Supplementary file 1 [file Data_Sheet_1.ZIP › Supplementary_Material/Supplementary figure.1.tif]

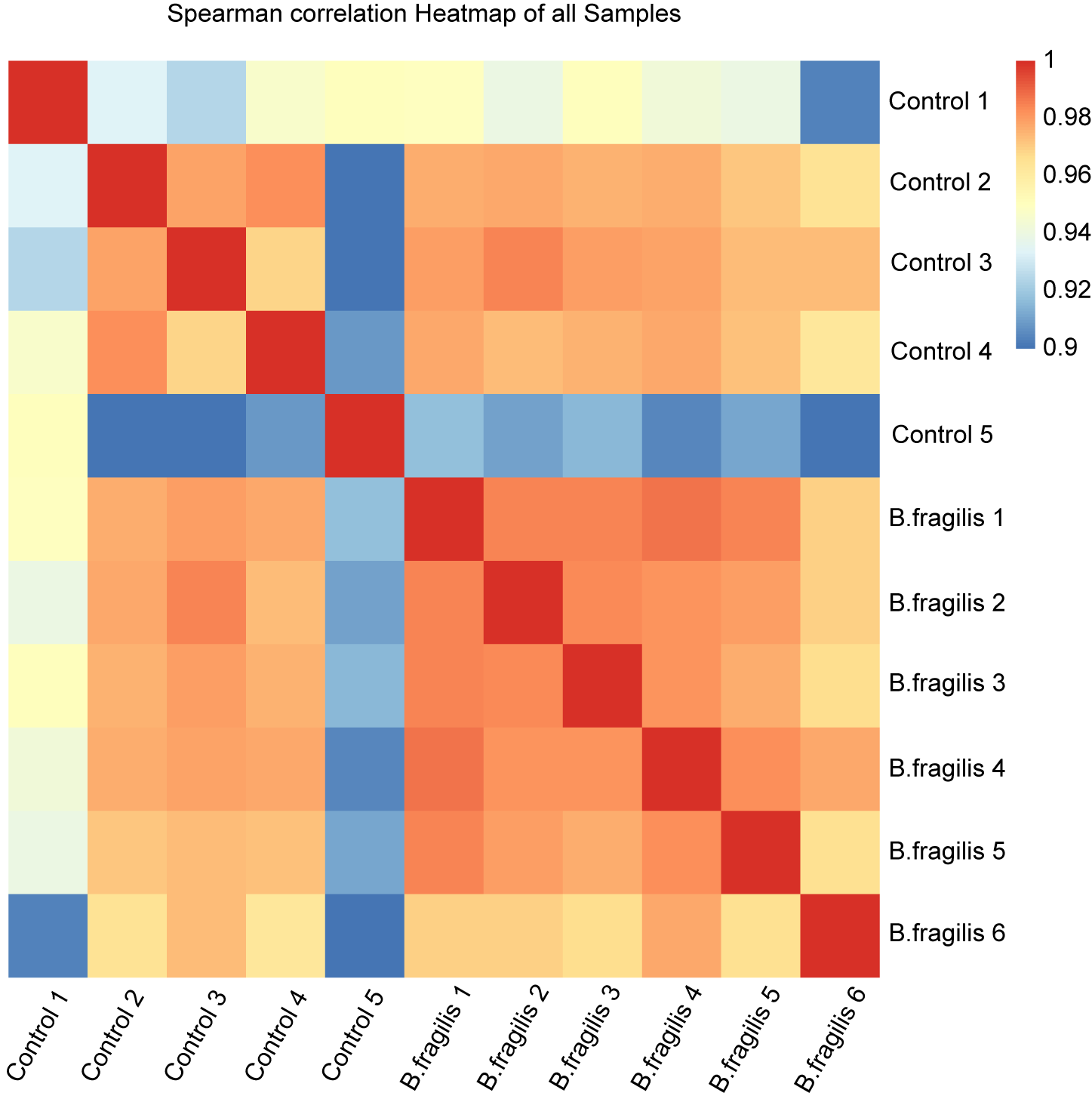

Supplement: Supplementary file 1 [file Data_Sheet_1.ZIP › Supplementary_Material/Supplementary figure.2.tif]

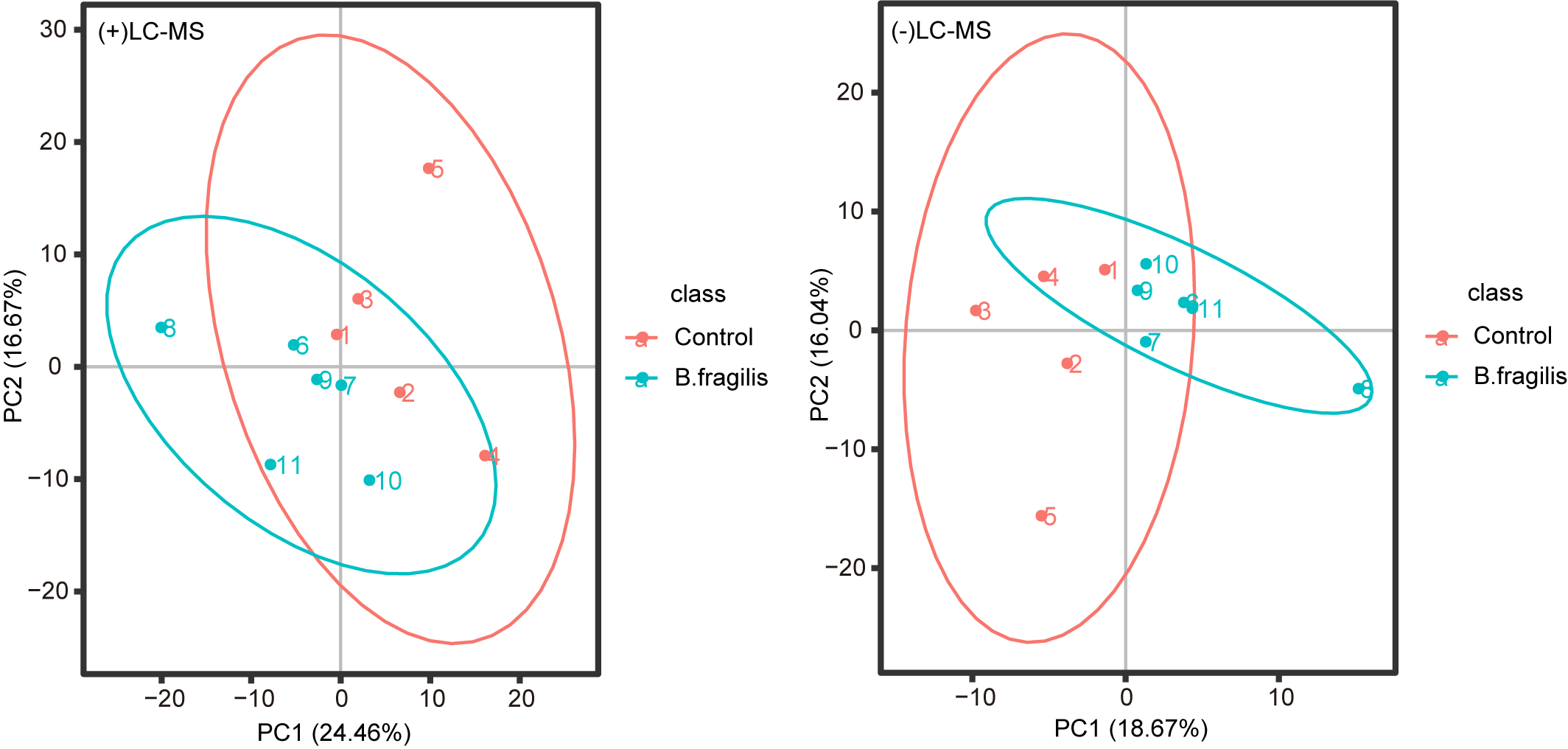

Supplement: Supplementary file 1 [file Data_Sheet_1.ZIP › Supplementary_Material/Supplementary figure.3.tif]
